# Quality of Life Impact of Velopharyngeal Insufficiency: The Role of Social Determinants of Health

**DOI:** 10.1002/lary.70189

**Published:** 2025-10-04

**Authors:** Wesley P. Allen, J. B. Eyring, Brandon M. Hemeyer, Reema Padia, Quinn T. Orb, Jeremy D. Meier

**Affiliations:** ^1^ Spencer Fox Eccles School of Medicine at the University of Utah Salt Lake City Utah USA; ^2^ Department of Otolaryngology—Head & Neck Surgery University of Utah Salt Lake City Utah USA; ^3^ Pediatric Otolaryngology Intermountain Health, Primary Children's Hospital Salt Lake City Utah USA

**Keywords:** caregiver perspective, social determinants of health, velopharyngeal insufficiency

## Abstract

**Objectives:**

The impact of velopharyngeal insufficiency (VPI) on patient and caregiver quality of life (QOL) is well documented. The social determinants of health (SDOH) that affect this relationship remain unclear. This study aimed to evaluate these associations to better understand how social context impacts patients and caregivers at risk of VPI due to congenital cleft and craniofacial deformities.

**Methods:**

Retrospective review of caregiver‐reported Velopharyngeal Insufficiency Effects on Life Outcome (VELO) questionnaire responses was conducted for patients seen in a multidisciplinary cleft and craniofacial clinic from 2020 to 2023. Scores were matched to census data regarding educational opportunities, health/environmental factors, and socioeconomic factors using the Childhood Opportunity Index (COI). Associations between QOL and SDOH were evaluated via linear regression, with higher scores representing better values.

**Results:**

Among the cohort (*N* = 161), multiple SDOH categories significantly predicted the QOL impacts of VPI (*p* < 0.05). Socioeconomic factors were positively correlated with speech limitations, situational difficulty, emotional impact, and caregiver impact (*β* = 0.23–0.36, *p* < 0.05). Contrary to our hypothesis, health/environmental factors exhibited a significant negative correlation across the same VELO domains in addition to swallowing problems (*β* = −0.29 to −0.12, *p* < 0.05). Educational opportunities showed no significant association with any VELO subcategory (*β* = −0.11 to −0.03, *p* > 0.1).

**Conclusion:**

Higher socioeconomic status was associated with better VELO scores, underscoring the protective role of resources in health outcomes and caregiver perceptions. In contrast, caregivers with better health/environmental conditions reported worse outcomes, suggesting that higher health standards may influence perceptions of VPI severity.

**Level of Evidence:**

3

## Graphical abstract

This study examined the association between social determinants of health and caregiver‐reported quality of life in children with velopharyngeal insufficiency (VPI). Higher socioeconomic status was associated with improved VELO scores, whereas more favorable health/environmental conditions correlated with worse outcomes. These findings underscore the nuanced influence of social context on patient and caregiver experiences with VPI.

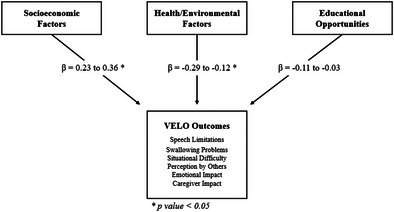

## 1 Introduction

Velopharyngeal Insufficiency (VPI) is a disorder that primarily affects pediatric populations and is generally secondary to craniofacial abnormalities, surgical complications, or genetic syndromes [1, 2, 3]. The impact of VPI on patients' speech, swallowing, and interactions with others can be extremely distressing to both children and caregivers, warranting further research and evaluation [4].

Social determinants of health (SDOH) have been studied in many aspects of medicine, highlighting important areas for intervention and resource development. Although a previous study by Li et al. examined the impact of select SDOH components on VPI outcomes, the analysis was limited by a relatively small study cohort and was only a minor focus of the study [5]. Additionally, although several studies have demonstrated the impact of SDOH on caregiver perception, this subject is relatively understudied within pediatric care in general and particularly within pediatric otolaryngology [6, 7, 8, 9, 10, 11].

The present study seeks to evaluate the impact of SDOH on VPI outcomes and caregiver perception by utilizing the Childhood Opportunity Index (COI) database in tandem with the Velopharyngeal Insufficiency Effects of Life Outcomes questionnaire. We hypothesized that higher (more favorable) SDOH scores across all domains would be associated with improved VPI outcomes and caregiver perceptions, highlighting potential VPI care gaps and identifying topics for future research within pediatric otolaryngology.

## 2 Methods

### 2.1 Participants and Procedures

Retrospective review was conducted of patients evaluated for VPI from January 2020 to December 2023 in the multidisciplinary cleft and craniofacial clinic at Primary Children's Hospital in Salt Lake City, Utah. All patients seen at this clinic have a diagnosed craniofacial anomaly, the most common being cleft lip or palate, along with other diagnoses including Goldenhar syndrome, Tessier cleft, among others. As these patients have an increased risk of VPI compared to the general population, caregivers of select patients (all those with a diagnosed cleft palate or with any of the aforementioned craniofacial anomalies and parent‐reported symptoms concerning VPI) were asked to complete a standardized questionnaire evaluating the impact of potential VPI‐related symptoms on their quality of life. This survey was administered to caregivers of these patients at their annual multidisciplinary team visit once the patient turned 5‐years old.

Inclusion criteria for the study were pediatric patients aged 5–18 who were seen in the clinic during the study period, had a caregiver‐completed questionnaire on file, and had a home address linkable to U.S. SDOH census data. For patients with multiple questionnaire responses available, the earliest recorded response was utilized for analysis.

### 2.2 Measures

The impact of VPI on quality of life was assessed by the Velopharyngeal Insufficiency Effects on Life Outcomes (VELO; Skirko et al.) questionnaire, a validated scale used to measure the quality of life impact of VPI on patients and their caregivers [12]. For this study, the caregiver version of the questionnaire was utilized in an attempt to best capture caregiver perception and was completed in clinic by caregivers present at their child's appointment. The VELO caregiver questionnaire includes 26 items across the following six subcategories with examples added for clarity: Speech Limitations (“Air comes out of his/her nose when talking” and “Difficulty speaking in long sentences”), Swallowing Problems (“Liquids come from the nose while drinking” and “Solid food comes from the nose while eating”), Situational Difficulty (“Speech is difficult for strangers to understand” and “Difficulty being understood on the phone”), Emotional Impact (“Teased because of speech” and “Child gets sad because of speech”), Perception by Others (“Treated as if not very bright because of speech” and “Others ignore my child because of his or her speech”), and Caregiver Impact (“I am worried about my child's speech” and “My child's speech problem slows me down”). Each item is rated on a 5‐point Likert scale ranging from 0 (Never) to 4 (Almost Always), over the past 4 weeks. Scores are then standardized, producing a numeric score between 0 and 100 with higher scores representing better outcomes.

SDOH were assessed by domains within the COI, including Socioeconomic Factors, Health/Environmental Factors, and Educational Opportunities [13, 14, 15]. The index components were linked to each patient by address using the Agency for Healthcare Research and Quality (AHRQ) SDOH database (based on 2020 census data). The AHRQ aggregates data from multiple sources to provide estimates that are comprehensive and representative. Socioeconomic Factors include economic stability, income levels, employment rate, and poverty rate. Health/Environmental Factors include air and water quality, access to healthcare facilities, prevalence of chronic diseases, and access to green space. Educational Opportunities include literacy rates, school funding, and educational attainment. Across all COI domains, higher scores represent more favorable environments within the category of interest.

### 2.3 Analysis

Descriptive and bivariate statistics were calculated for demographic and baseline characteristics (Table 1). Linear regression modeling assessed relationships between SDOH and VPI impact on quality of life (Table 2). All data were analyzed using SAS version 9.

**TABLE 1:** Bivariate statistics of measures and descriptive statistics.

	*n*	*M*	SD	Range	1	2	3	4	5	6	7	8	9	10	11
Childhood Opportunity Index															
1. Overall COI Score	161	67.9	17.7	1–99	—	—	—	—	—	—	—	—	—	—	—
2. Educational opportunities	161	54.3	23.8	1–98	0.81**	—	—	—	—	—	—	—	—	—	—
3. Health/environmental factors	161	70.6	19.0	9–98	0.63**	0.38**	—	—	—	—	—	—	—	—	—
4. Socioeconomic factors	161	71.8	17.9	2–99	0.95**	0.62**	0.55**	—	—	—	—	—	—	—	—
VELO questionnaire															
5. Overall VELO score	161	78.1	19.9	0–100	0.07	0.04	−0.01	0.11	—	—	—	—	—	—	—
6. Speech limitations	161	79.3	17.6	0–100	0.01	−0.06	−0.13*	0.08	0.77**	—	—	—	—	—	—
7. Swallowing problems	161	70.5	26.1	0–100	0.01	0.01	−0.11	0.06	0.76**	0.79**	—	—	—	—	—
8. Situational difficulty	161	77.0	22.0	0–100	0.06	0.02	−0.11	0.12	0.78**	0.83**	0.87**	—	—	—	—
9. Emotional impact	161	90.2	15.9	0–100	0.03	−0.03	−0.056	0.10	0.69**	0.73**	0.59**	0.74**	—	—	—
10. Perception by others	161	82.4	20.1	0–100	−0.01	−0.05	−0.14*	0.06	0.75**	0.75**	0.81**	0.79**	0.69**	—	—
11. Caregiver impact	161	81.7	20.5	0–100	−0.01	−0.06	−0.15*	0.06	0.72**	0.73**	0.79**	0.78**	0.64**	0.97**	—
**Patient age**	161	9.8	3.2	5–17	−0.03	−0.02	0.05	−0.04	−0.06	−0.11	0.04	0.03	−0.10	−0.05	−0.07

*Note*: Values represent Pearson's Correlation Coefficients.

Abbreviations: *M* = mean; SD = standard deviation.

* *p* < 0.1.

** *p* < 0.001.

**TABLE 2:** Standardized beta coefficients for the effects of three Childhood Opportunity Index subscales on VELO outcomes, controlling for patient age.

Outcome	Predictor	*β*	SE	*t*	*p*
Speech limitations	Educational opportunities	−0.11	0.07	−1.47	0.14
	Health and environmental factors	−0.21	0.09	−2.46	0.02
	Socioeconomic factors	0.28	0.11	2.61	< 0.01
	Patient age	−0.50	0.44	−1.16	0.25
Swallowing problems	Educational opportunities	−0.03	0.12	−0.26	0.79
	Health and environmental factors	−0.28	0.13	−2.16	0.03
	Socioeconomic factors	0.27	0.16	1.66	0.09
	Patient age	0.49	0.65	0.75	0.46
Situational difficulty	Educational opportunities	−0.06	0.09	−0.65	0.51
	Health and environmental factors	−0.29	0.11	−2.66	< 0.01
	Socioeconomic factors	0.36	0.14	2.68	< 0.01
	Patient age	0.36	0.54	0.65	0.51
Perception by others	Educational opportunities	−0.11	0.08	−1.32	0.19
	Health and environmental factors	−0.24	0.09	−2.42	0.01
	Socioeconomic factors	0.29	0.12	2.37	0.02
	Patient age	−0.17	0.50	−0.33	0.74
Emotional impact	Educational opportunities	−0.10	0.07	−1.47	0.14
	Health and environmental factors	−0.12	0.08	−1.48	0.14
	Socioeconomic factors	0.23	0.09	2.38	0.02
	Patient age	−0.45	0.39	−1.14	0.26
Caregiver impact	Educational opportunities	−0.12	0.08	−1.46	0.15
	Health and environmental factors	−0.26	0.10	−2.6	< 0.01
	Socioeconomic factors	0.32	0.12	2.58	0.01
	Patient age	−0.32	0.50	−0.63	0.53

Abbreviation: SE = standard error.

This study was approved by the Institutional Review Board of the University of Utah and Intermountain Health's Primary Children's Hospital. A waiver of informed consent was granted due to the retrospective design and minimal risk to participants.

## 3 Results

A total of 161 patients (*n* = 161) met the inclusion criteria, with 61.5% (*n* = 99) identifying as male, 37.9% (*n* = 61) as female, and 0.6% (*n* = 1) with unreported gender; ages ranged from 5 to 17 with an average age of 9.8 (see Table 1). Patient‐reported race/ethnicity was not analyzed as this was inconsistently reported within patient charts. However, within the census tracts of interest, the pediatric population was 76.33% (SD = 15.18%) White, 1.15% (SD = 1.72%) Black, 15.48% (SD = 11.47%) Hispanic, 1.72% (SD = 7.04%) American Indian or Alaskan Native, 2.07% (SD = 2.34%) Asian or Pacific Islander, and 9.41% (SD = 8.22%) of two or more races.

Significant positive correlations were seen between all COI subscales as seen in Table 1 (*p* < 0.001). VELO Questionnaire subcategories also showed strong intercorrelations, with positive relationships seen between all VELO domains (*p* < 0.001). Notable correlations were observed between Health/Environmental Factors and Speech Limitations (*r* = −0.13, *p* = 0.09), Perception by Others (*r* = −0.13, *p* = 0.08), and Caregiver Impact (*r* = −0.14, *p* = 0.06). Patient age was not associated with COI or VELO measurements in both the Pearson's Correlation Coefficients and the Linear Regression model.

**FIGURE 1:**
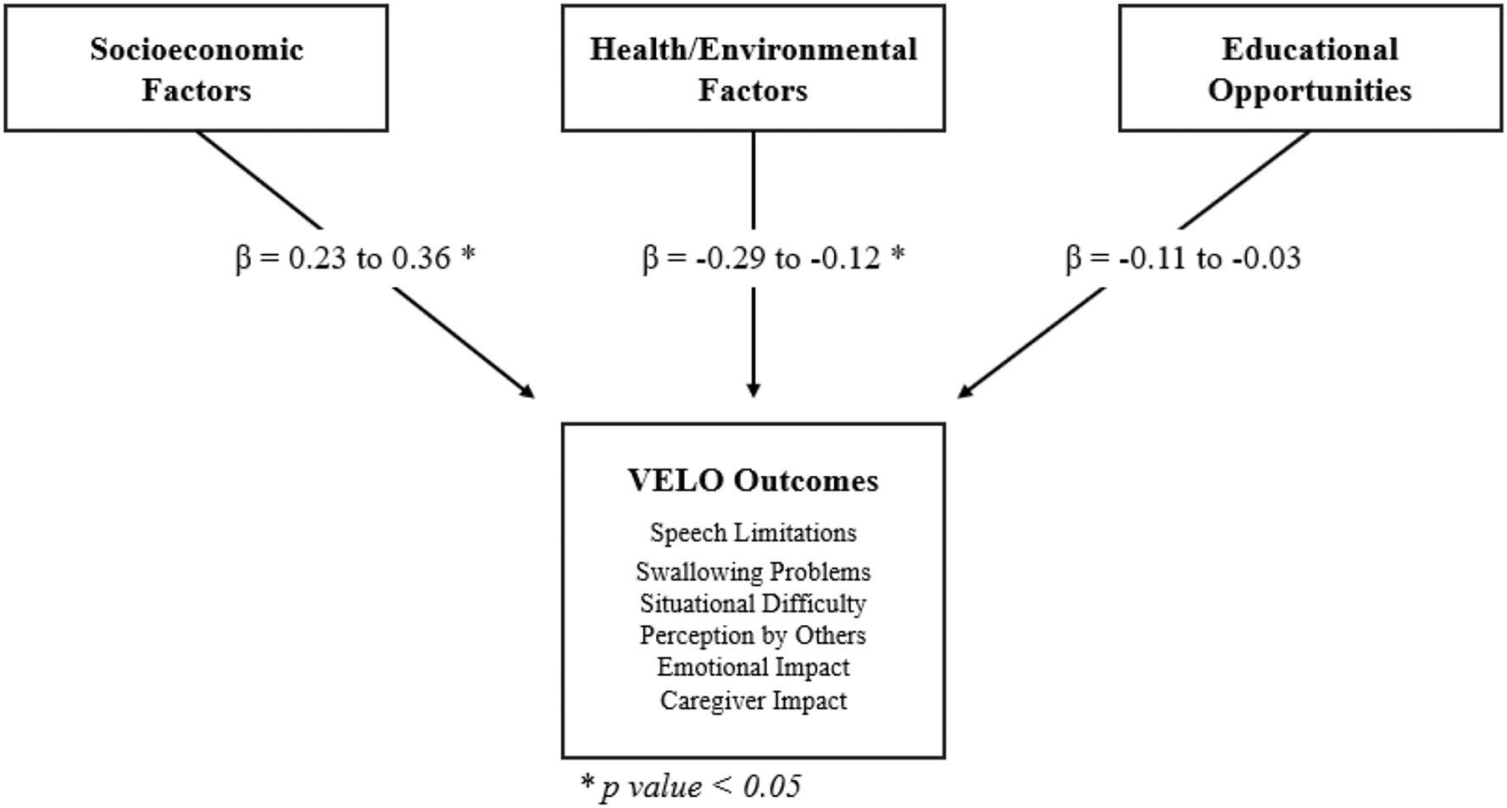
Socioeconomic factors showed a significant positive correlation with all VELO subcategories except Swallowing Problems (*p* = 0.09), suggesting caregivers with more resources reported better VPI‐related quality of life. Contrary to our hypothesis, Health/Environmental factors showed significant negative correlations across all subcategories except Emotional Impact (*p* = 0.14), indicating caregivers in better health and environmental conditions generally reported poorer outcomes than those in areas with worse conditions. Educational opportunities did not significantly correlate with any VELO subcategories.

Socioeconomic Factors showed significant positive associations with Speech Limitations (*β* = 0.28, *p* < 0.01), Situational Difficulty (*β* = 0.36, *p* = 0.04), Perception by Others (*β* = 0.29, *p* = 0.02), Emotional Impact (*β* = 0.23, *p* = 0.02), and Caregiver Impact (*β* = 0.26, *p* = 0.03), suggesting higher socioeconomic status was associated with improved outcomes (see Table 2). Positive but non‐significant associations were also noted between Socioeconomic Factors and Swallowing Problems (*β* = 0.27, *p* = 0.09). Conversely, Health/Environmental Factors displayed significant negative associations with Speech Limitations (*β* = −0.21, *p* = 0.02), Swallowing Problems (*β* = −0.28, *p* = 0.03), Situational Difficulty (*β* = −0.29, *p* < 0.01), Perception by Others (*β* = −0.24, *p* = 0.01), and Caregiver Impact (*β* = −0.26, *p* = 0.03), indicating that better health and environmental conditions were associated with poorer outcomes. Negative but non‐significant associations were also noted between Health/Environmental Factors and Emotional Impact (*β* = −0.12, *p* = 0.14). Educational Opportunities showed no significant associations with any VELO subcategories, with Speech Limitations (*β* = −0.11, *p* = 0.14), Emotional Impact (*β* = −0.10, *p* = 0.14), and Caregiver Impact (*β* = −0.12, *p* = 0.15) closest to significance. A summary of the associations across all three COI domains is shown in Figure 1.

## 4 Discussion

In this study assessing parent‐reported VELO scores in children at high risk for VPI, SDOH, specifically socioeconomic factors and health/environment factors, were associated with reported VELO outcomes. Higher Socioeconomic Factors scores were consistently associated with better scores across nearly all VELO subcategories, suggesting that caregivers with more socioeconomic resources were more likely to report better quality of life outcomes regarding their children's VPI. These findings align with past research indicating that socioeconomic resources play a protective role in health outcomes, while further building upon the established literature by highlighting the breadth and complexity of this association across caregivers' experience [16, 17, 18, 19]. Contrary to our initial hypothesis, caregivers in areas with better health and environmental conditions generally reported poorer outcomes than those in areas with worse conditions, highlighting the complex nature of health perceptions and resource utilization. While this finding was largely unexpected, it offers insights into factors that may influence caregivers' perception of pediatric health conditions, highlighting areas of future research and intervention. Educational Opportunities did not significantly correlate with caregiver perceptions across any of the VELO subcategories investigated.

### 4.1 Socioeconomic Factors

The positive correlation between socioeconomic status and VELO outcomes is likely multifactorial, with all VELO subcategories likely affected by lack of healthcare access and limited resources [20, 21, 22, 23, 24, 25]. As speech therapy often requires significant out‐of‐pocket expense, this association may be explained by patients with higher socioeconomic status having better access and increased funds to afford speech therapy. Speech Limitations and Situational Difficulty are likely intertwined, as the Situational Difficulty domain includes questions regarding a child's ability to articulate thoughts during social interactions. Speech therapy may be beneficial for both of these VELO domains and may represent an area of inequity due to limited insurance coverage or inability to afford regular visits [20, 26, 27, 28]. Additionally, patients with limited socioeconomic resources encounter increased access barriers to both primary care providers and specialists, subsequently delaying diagnosis and referral to relevant care providers [29, 30, 31]. The impact of Socioeconomic Factors on Emotional Impact and Perception by Others may result from higher rates of depression and anxiety in populations with fewer resources [32, 33]. These patterns are seen in both children and adults, suggesting that the emotional burden seen in this study may reflect both the true emotional toll of VPI on the children and the emotional distress of the caregivers responding to the questionnaire [34, 35]. Poorer Caregiver Impact scores likely reflect similar stressors along with the increased strain on these caregivers, including higher rates of single‐parent homes, reduced social support, and limited financial flexibility [36, 37, 38, 39]. Decreased health literacy may also contribute, leading to poor adherence and inaccurate expectations [40, 41, 42, 43].

When addressing the multifaceted inequities facing patients from low socioeconomic backgrounds, physicians should consider a variety of approaches that address the complex needs of this patient population. Increasing patient access to VPI care may be improved by strengthening connections between primary care providers and pediatric otolaryngologists. Family medicine practitioners, pediatricians, and obstetricians often serve as patients' initial point of contact within the healthcare system [30, 44, 45]. By enhancing these providers' understanding of conditions leading to VPI (both prenatally and within pediatric care), symptoms can be identified earlier, leading to better outcomes. As the patients in this study were all seen in a multidisciplinary cleft program, cleft team providers should recognize that children with lower socioeconomic status may be at higher risk of VPI. Addressing caregiver burnout is also important, given VPI's extended course and the complex multidisciplinary teams involved. Community health workers and social workers may be utilized to assess patients' social context (e.g., food insecurity, housing stability, etc.) and create care plans mindful of caregivers' energy and resources [46, 47]. Coordinating care across specialties (e.g., scheduling same‐day appointments) and increasing the use of telehealth may reduce caregiver disruption and transportation costs [48, 49]. Health literacy interventions may also be helpful, improving adherence and setting appropriate expectations throughout treatment [50].

### 4.2 Health/Environmental Factors

The negative associations between Health/Environmental Factors and VELO outcomes suggest that caregivers in favorable conditions may report worse outcomes. This counterintuitive result may reflect underlying complexities in how these factors affect caregiver perceptions. Patients in healthier neighborhoods are more likely to access healthcare and compare their health to healthier social circles, leading to greater awareness of deficiencies [51, 52]. It is reasonable to hypothesize that caregivers in areas with better resources will subsequently have higher health standards for their children and are thus more likely to report worse VELO outcomes than those in areas where health is a lower priority. As a result, children living in areas with limited health resources may be inadvertently underdiagnosed and subsequently undertreated, highlighting the importance of thorough clinical evaluation and the use of additional objective measurements throughout treatment. Additionally, establishing proper expectations with caregivers early on in the treatment course may prevent problems from going unreported and allow for more equitable care across diverse patient populations [53, 54].

It is important to note that the negative correlation seen between SDOH and reported VELO outcomes does not diminish the importance of investing in community resources that promote healthier communities and prioritize safe environments. Although improving access to healthcare and increasing health education may inadvertently increase reported health concerns, this may be due to an elevated awareness of existing conditions rather than an actual rise in health deficiencies [55, 56]. By increasing awareness, caregivers may be more inclined to obtain needed medical care, leading to improved long‐term health outcomes for their children [57, 58]. Prioritizing caregiver education in areas with large pediatric populations can establish healthier standards of living for young patients, leading to habits that can persist throughout their lifetimes and lead to healthier communities [59, 60].

### 4.3 Educational Opportunities

The lack of relationship between Educational Opportunities and VELO Scores is unclear, but may reflect the current role of schools in recognizing VPI among other developmental barriers [61, 62, 63]. While many disorders can be identified within the school setting, VPI is relatively uncommon, making it difficult for educators to recognize symptoms and connect caregivers with appropriate resources [64, 65, 66]. Thus, the absence of correlation may indicate a general lack of VPI‐related resources across all school systems rather than equal availability of adequate resources for all populations. Future research is warranted to evaluate ways to improve VPI identification within the school setting, as this may be a largely untapped resource in most areas.

## 5 Limitations

The single‐institution cohort may not reflect broader populations in terms of socioeconomic and racial diversity. As such, the patients included in this study may not fully represent the socioeconomic breadth, racial/ethnic diversity, or environmental conditions of the cleft and craniofacial population across the country, which may influence these results. Additionally, the retrospective design of this study limits its generalizability, as it restricts the ability to control for potential confounding factors, such as caregiver demographics, health literacy, and baseline mental health status, all of which may influence both caregiver perceptions and reported outcomes. These findings should therefore be applied to other institutions with caution.

While these data offer insights into the role of Socioeconomic Factors in VPI, the patient group investigated is not a random sample of all individuals with this condition, but rather a select group of children with orofacial cleft deformities that are at risk of VPI. Although the cohort includes patients from various socioeconomic backgrounds (Socioeconomic Factors range of 2–99, Table 1), individuals with low socioeconomic status were likely underrepresented due to access barriers. As such, findings represented in this study may underestimate the impact of Socioeconomic Factors on VELO scores. Caution should be taken before discounting the association between Socioeconomic Factors and Swallowing Problems (*β* = 0.27, *p* = 0.09), as a stronger relationship may appear if all affected patients were included. Similarly, the lack of correlation between Educational Opportunities and VELO outcomes may be due to limited representation, as the quality of public education in the study region is generally at or above the national average [67]. Further research is warranted to fully elucidate the impact of educational resources on VPI outcomes.

The patients seen at the multidisciplinary cleft and craniofacial clinic are typically seen annually. However, some children present at different timelines or are seen after previous treatment from surgeons outside of the team. As such, it is difficult to capture VELO scores that are representative of a single, common timepoint across all patients. While these findings still shed light on important topics for individuals caring for VPI patients, further research, and particularly prospective studies, would be beneficial to better understand the relationship between SDOH, treatment patterns, and VPI outcomes.

Variation in cleft subtype and the presence of underlying syndromes likely influence both patient and caregiver experiences, which may be reflected in reported VELO scores. Although this study does not stratify findings based on concurrent diagnoses or specific cleft characteristics, further research is warranted to evaluate how these factors may impact outcomes for patients and their families.

The VELO questionnaire is subjective and cannot directly measure VPI severity. Further research is needed to objectively assess the impact of Socioeconomic Factors, Health/Environmental Factors, and Educational Opportunities on VPI outcomes. Correlating the findings within this study with objective data such as nasometry scores, perceptual speech assessments, and swallowing studies may provide further insight and allow for a more robust analysis. Additionally, although the VELO questionnaire covers common VPI complications, future studies using direct interviews with caregivers and patients may be helpful in identifying additional care gaps.

## 6 Conclusion

This study highlights the complex interplay between socioeconomic factors, health/environmental conditions, and VELO outcomes in children at risk of VPI. Higher socioeconomic status was consistently associated with better VELO scores, underscoring the protective role of resources in health outcomes and caregiver perceptions. However, contrary to expectations, caregivers in areas with better health and environmental conditions reported worse outcomes, suggesting that higher health standards and greater awareness may influence perceptions of VPI severity. The lack of correlation between educational opportunities and VELO scores may reflect the challenges schools face in identifying and addressing VPI, pointing to a potential gap in resources.

In clinical practice, pediatric otolaryngologists should identify ways to strengthen their connections with primary care providers, improve health literacy in areas with large pediatric populations, and consider social context when addressing caregiver burnout. Future research should focus on objectively assessing the impact of socioeconomic and environmental factors on VPI outcomes and exploring ways to improve early identification and intervention, particularly with those of lower socioeconomic status.

## Ethics Statement

This study was approved by both the Primary Children's Hospital and University of Utah's Institutional Review Board.

## Conflicts of Interest

The authors declare no conflicts of interest.

## Data Availability

The data that support the findings of this study are available on request from the corresponding author. The data are not publicly available due to privacy or ethical restrictions.
